# Visual distraction during word-list retrieval does not consistently disrupt memory

**DOI:** 10.3389/fpsyg.2014.00362

**Published:** 2014-04-23

**Authors:** Pamela J. L. Rae, Timothy J. Perfect

**Affiliations:** School of Psychology, University of PlymouthPlymouth, UK

**Keywords:** visual distraction, dynamic visual noise, episodic memory, word-list recall, recall error, embodied memory

## Abstract

Glenberg et al. (1998) reported that episodic memory is impaired by visual distraction and argued that this effect is consistent with a trade-off between internal and external attentional focus. However, their demonstration that visual distraction impairs memory for lists used 15 consecutive word-lists, with analysis only of mid-list items, and has never been replicated. Experiment 1 (*N* = 37) replicated their methodology and found the same pattern of impairment for mid-list recall, but found no evidence of impairment for other items on the lists. Experiment 2 (*N* = 64) explored whether this pattern arises because the mid-list items are poorly encoded (by manipulating presentation rate) or because of interference. Experiment 3 (*N* = 36) also looked at the role of interference whilst controlling for potential item effects. Neither study replicated the pattern seen in Experiment 1, despite reliable effects of presentation rate (Experiment 2) and interference (Experiments 2 and 3). Experiment 2 found no effect of distraction for mid-list items, but distraction did increase both correct and incorrect recall of all items suggestive of a shift in willingness to report. Experiment 3 found no effects of distraction whatsoever. Thus, there is no clear evidence that distraction consistently impairs retrieval of items from lists and therefore no consistent evidence to support the embodied cognition account used to explain the original finding.

## INTRODUCTION

The physical environment is often distracting. Open-plan work places, for example, are replete with visual and auditory background noise: 99% of office workers responding to Banbury and Berry’s (2005) survey claimed that this noise was so distracting it adversely affected concentration. Considering that it is commonplace to carry out daily tasks in distracting environments, it is not surprising that numerous researchers have investigated the effect of distraction on cognitive processes including episodic memory.

Evidence that environments are distracting to retrieval processes comes from observations of gaze aversion. When trying to remember an item from memory, people often look away from their immediate environment in order to suppress its distracting effect (Doherty-Sneddon and Phelps, 2005). For example, (Glenberg et al. (1998; Experiment 1) observed that participants were increasingly likely to avert their gaze during recall the further the target memories were back in time. This suggests that as the task becomes more difficult, people spontaneously avert their gaze away from the distracting environment in order to focus attention inwardly to the task of retrieval. Although gaze aversion is also commonly seen during social interactions (Kendon, 1967) two studies suggest that it serves more of a distraction-suppression function than a social function. Glenberg et al. (1998; Experiment 3) video-taped participants whilst they sat alone in a laboratory typing answers to increasingly difficult general knowledge questions. In the absence of any social interaction the frequency of gaze aversion increased as memory task-difficulty increased. Doherty-Sneddon and Phelps (2005) found that regardless of whether the interview was conducted in person or by video-link-up, the frequency of gaze aversion was driven by the difficulty of the memory retrieval task, rather than the interview setting. In contrast to these findings, Markson and Paterson (2009; Experiment 2) found that performance on a visual-spatial imagination task was poorer when participants maintained face-to-face eye-contact with the experimenter compared to when averting their gaze by looking at a photograph of the experimenter or closing their eyes. Although the authors conclude that the benefits of gaze aversion are a result of removing the face-to-face social aspect of eye-contact, they are clear to point out that these findings are based on performance of a visual-spatial imagination task which, unlike the above two studies, does not involve memory recall.

Additional evidence of the distracting nature of the environment comes from the field of eye-witness interviews which has looked at the beneficial effects of reducing environmental distraction via instructed eye-closure (EC), and the negative effects of experimental increases in environmental distraction. Wagstaff et al. (2004; Experiment 2) asked participants to recall details of a prominent past event with their eyes open or their eyes-closed. Their participants had all watched the live television broadcast of Diana, Princess of Wales’s funeral some 5 years earlier but had not watched it again since. Participants answered a set of questions about the event under instructions to keep their eyes open or closed. Instructed EC led to more correct answers (*d* = 0.57), with no difference in the rate of wrong answers. Perfect et al. (2008) investigated the effect of EC compared to a no-instruction control group in a series of five experiments which varied the nature of the event witnessed (a video-clip or live event) and the recall task (cued recall or free-narrative account). In all studies there was a benefit of instructed EC on recall of correct details with an (un-weighted) average effect size of *d* = 0.98. Instructed EC also led to a decrease in the number of incorrect details recalled, with an (un-weighted) average effect size of *d* = -0.34. In all studies, participants were free to withhold responses (i.e., say “don’t know” to a question, or withhold a detail in free report), but EC had no impact upon willingness to provide an answer. Instead, EC increased the *accuracy* of what was reported. Beneficial effects of EC have also been reported for videos of violent events (Vredeveldt et al., 2011), for increasing correct recall of coarse-grain visual and auditory details of a violent video-clip and for decreasing incorrect recall of visual details (Vredeveldt et al., 2012), with a delay of 1 week prior to test (Vredeveldt et al., 2013), when there is a shift in context between event and test environment (Vredeveldt and Penrod, 2013) and with child witnesses (Mastroberardino et al., 2012).

Another line of research has manipulated levels of environmental distraction during retrieval. Perfect et al. (2012) manipulated the amount of visual distraction during retrieval of details about a videotaped event. Distraction took the form of colored squares changing location (to one of the four corners of the screen) every 1.5 s. In the simple distraction conditon a single box moved, whilst in the complex condition two (differently colored) boxes moved simultaneously. Increased distraction did not alter willingness to answer but it led to fewer correct and more incorrect answers, with large effect sizes of *d =* 2.05 and *d* = -1.78, respectively. Vredeveldt et al. (2011) manipulated visual and auditory distraction during retrieval under four conditions: participants were presented with a stream of Hebrew words which either appeared in random locations on a screen or were spoken out aloud (both high distraction) or were asked to close their eyes or look at a black screen (both low distraction). Once again, there was no difference in participants’ willingness to answer a question but high distraction led to fewer correct and more incorrect answers (effect sizes, *d* = 0.48, *d* = -0.40, respectively). Two studies looking at the effect of distraction on visual memory (Wais et al., 2010; Wais and Gazzaley, 2011) also found that distraction decreases retrieval-accuracy. In both, participants studied images of objects appearing either singularly or in multiples (up to four of the same object on the same image slide) and were later given a verbally presented memory test in which participants had to say how many exemplars they had seen previously (0 for new items, or 1–4 for items shown previously). Both studies reported that visual and auditory distraction (participants looked at a picture of an outdoor scene or listened to pre-recorded noise from a restaurant) reduced the accuracy of the judgment of how many exemplars had previously been presented (average effect size of *d* = 0.50). Perfect et al. (2011) examined both environmental distraction and the potential benefit of EC. Participants answered questions about a staged event in conditions of quiet, or with white noise as distraction, either with instructed EC, or a no-instruction control. The effects of the white noise were to increase the rate of wrong answers provided, but this effect was reduced in participants instructed to close their eyes.

Thus there is a fairly clear pattern of effects for environmental distraction (or its removal though EC): increasing the level of distraction in the environment decreases retrieval-accuracy, often without changing response bias, whilst suppressing the influence of the environment through instructed EC increases retrieval-accuracy, even compared to a no-instruction control that may involve some EC or gaze aversion.

Surprisingly, given the ubiquity of studies of verbal memory, only one study has reported the simple effects of environmental distraction at retrieval on recall of words studied in lists. Glenberg et al. (1998) widely cited and influential paper reported a series of studies looking at gaze aversion and EC and also, looked at simple distraction effects on word-list recall.

In Experiment 5 of Glenberg et al. (1998) participants were presented with a total of ten 15-word word-lists, each followed by a 20 s arithmetic filler task, followed by a 30 s verbal recall period for the list items. During this participants either looked at a screen showing a picture of a sunset (static distraction) or watched a silent movie-clip from a Charlie Chaplin film (dynamic distraction). However, somewhat unexpectedly, the authors do not report recall performance for all target items in a list, and nor do they report the effect of list order: instead they report the effects of distraction only for the middle five words from each list, averaged over the 10 word-lists. This analysis revealed that distraction slightly reduced correct recall of mid-list items (*d* = 0.29: a small to medium effect size, Cohen, 1992).

**FIGURE 1:**
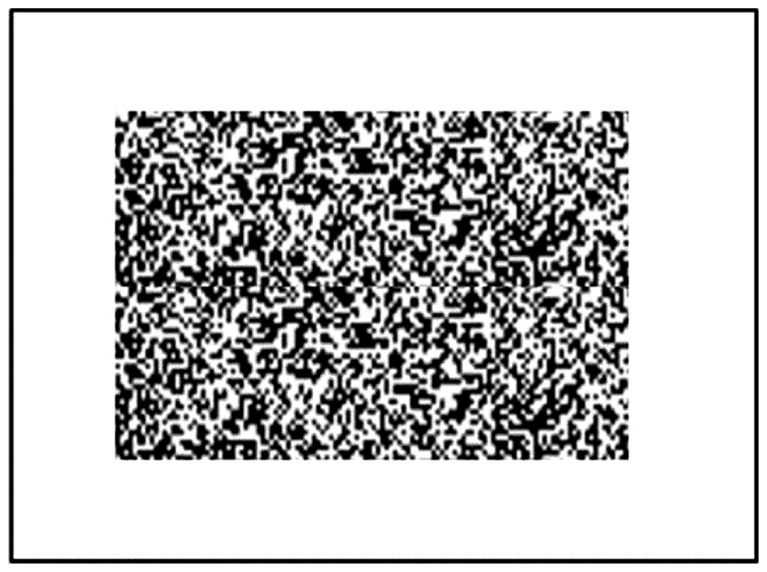
**Static visual noise (SVN), a screen of random black and white squares**. Dynamic visual noise (DVN) is achieved by changing black boxes (10 × 10 pixels) to white and vice versa at a rate of 291 per second.

Thus, despite being widely cited, the one study to look at environmental distraction effects on list recall found only a small effect, under highly atypical conditions: when multiple similar lists were studied, with only the mid-list items compared, and with a manipulation of distraction that compared a static photograph with a silent movie. What is unclear is the role of these factors in producing the effect, and thus the replicability and generalizability of the effect. Consequently, we set out to replicate Glenberg et al. (1998) Experiment 5 with a more closely controlled manipulation of distraction, and with analyses that looked at recall for all items on the list. The broader issue of our research is to further understand memory by investigating the mechanism by which environmental distraction disrupts memory recall. We used dynamic visual noise (DVN) as our distraction stimuli, and contrasted the effects of DVN with a static version of a DVN stimulus, which we hereafter refer to as static visual noise (SVN). **Figure 1** provides an example SVN image, along with the details of the nature of DVN. The negative effects of DVN have been reported in several memory studies: when words are recalled using a pegword mnemonic, (McConnell and Quinn, 2000; Andrade et al., 2002; Quinn and McConnell, 2006), when identifying visual changes in patterns (Dean et al., 2008), and when high imagery words rather than low imagery words are recalled (Parker and Dagnall, 2009). The aim of Experiment 1 was simply to replicate Glenberg et al. (1998) Experiment 5 as closely as possible, with a semantically neutral form of distraction, and then to analyse the data more thoroughly in order to determine the generality of any distraction effect. As effects of EC on word-list recall have not yet been explored, we also included a third condition where participants were instructed to close their eyes during word-list recall. However, as we explain below, we were not able to analyse these data.

## EXPERIMENT 1

The following three Experiments were carried out in line with ethical standards as set out by the University of Plymouth, School of Psychology ethics committee. Throughout the following analyses an alpha level of 0.05 was used, however, we further explored any numerical trends where 0.05 < *p* < 0.075.

### METHOD

#### Participants

Thirty-nine participants (24 females), average age 25.9 years (*SD* = 9.33) took part for course credit or as a paid volunteer. All participants had normal or corrected to normal vision and were fluent English speakers. All participants were made aware that the study involved being exposed to onscreen flickering; anyone concerned about this effect or with a history of seizures or migraines was excluded from the study. One participant’s data (male, aged 28 years) was excluded from analysis due to failure to comply with procedural instructions (consistently looking away from the visual distractor) and another (female, aged 20 years) was incomplete due to being interrupted by a fire-alarm.

#### Materials

***Word-lists***. One-hundred and fifty words were randomly selected from the 1,080-word Toronto Word Pool (Friendly et al., 1982). This selection was used to randomly generate (without replacement) a unique set of 15 lists of 15-words for each participant.

***Filler task***. A pool of 150 two-addend addition sums (e.g., 24 + 3 = ) was created from which 15 sets of 10 sums were randomly selected without replacement, for each participant.

***Distraction conditions***. Static visual noise and DVN were presented on a computer screen using parameters set out by McConnell and Quinn (2000): each field measured 700 × 700 pixels and consisted of a random pattern of 10 × 10 pixel blocks of black and white squares. This field was static during the SVN condition but appeared to flicker during the DVN condition as random pixel blocks changed color from black to white to black at a rate of 291 per second (see **Figure 1**). The surrounding background screen was white. A third condition of EC was also included and during the recall period under this condition, the program displayed a blank white screen for the entire recall period. The order in which SVN, DVN and EC conditions were presented was randomized across the 15-word-lists.

#### Procedure

Participants studied lists of individual words presented visually for 2 s each, with an inter-stimulus interval of 150 ms. Words were centered in the middle of the screen and appeared in black capital Arial-font, size 18. A series of 10 sums immediately followed the presentation of each word-list; each sum was shown center screen for 2 s at a time with a 200 ms inter-stimulus interval between sums. Participants were asked to verbally provide the solution to each sum as it appeared on the screen but were informed that answers were not being recorded or scored. All participants answered all sums. Following the last sum an onscreen instruction asked participants to either “Keep looking at the screen” (for SVN and DVN conditions) or informed them that they should keep their “Eyes-closed”; this instruction remained for 2 s and was followed by a fixed 30 s recall period. During the fixed recall period, participants verbally recalled words from the word-list they had just seen whilst looking at a screen which displayed SVN or DVN for the entire 30 s, or keeping their eyes-closed. Prior to the start of the experiment participants were informed that the experimenter would check to see if they complied with the instructions to look at the screen or close their eyes; although it was rarely required, a verbal reminder was given when necessary. The experimenter was seated adjacent to participants such that participants were unable to make eye-contact with the experimenter during encoding or retrieval phases. Across all participants, four words were recalled outside the 30 s recall period and these were excluded from analysis. Each participant recalled from five word-lists under SVN, five under DVN, and five under EC instructions in a randomized order: participants were not aware which recall condition would be used until after the word-list had been presented. At the end of each fixed recall period, which was signaled by a tone, participants pressed the space bar when ready to continue to the study phase for the next word-list.

### RESULTS AND DISCUSSION

Our first analysis was designed to replicate the mid-list analysis reported in Glenberg et al. (1998). Additionally, because we were interested in the generality (vs. specificity) of any distraction effect across items, we also looked at the effects of distraction on recall for items from the start and end of each list. Consequently, we ran the analysis of recall split by third of list (first-, mid-, and last-5 items for each list). We had also intended to explore whether instructed EC improved recall. However, upon inspection of the data from the EC condition it became clear that a coding error in the program had resulted in the EC condition being not properly counterbalanced across list order, and so we dropped this condition from the analysis. This error did not affect the comparison of the DVN and SVN conditions. Further details are available from the first author upon request.

Throughout all the following analyses, Greenhouse–Geisser adjustments are reported wherever Mauchley’s test of sphericity was significant.

**FIGURE 2:**
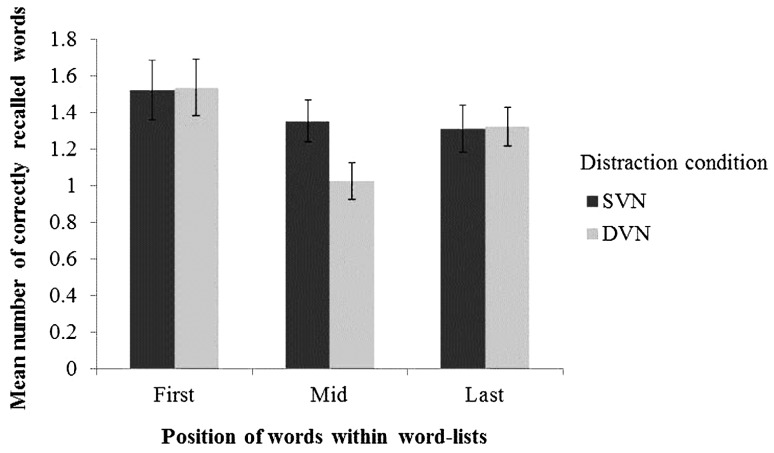
**The mean number of correctly recalled words from the First-, Mid-, and Last- five positions of each list.** Error bars represent standard errors of the mean.

A 3(Word Position: recall from first 5, middle 5, last 5 items in each list) × 2(Distraction: DVN vs. SVN) repeated measures ANOVA on correct recall showed there were no difference between the number of words recalled from the mid-list position of word-lists than from the first- and last-list positions, *F*(1,36) = 2.40, *p* = 0.098, MSE = 5.99, partial η^2^ = 0.063 and nor was there a difference between the overall number of words recalled under DVN compared to SVN, *F*(1,36) = 1.59, *p* = 0.215, MSE = 6.25, partial η^2^ = 0.042. However, as illustrated in **Figure 2**, fewer mid-list words were recalled under DVN than SVN, *F*(2,72) = 7.90, *p* = 0.001, MSE = 7.171, partial η^2^ = 0.18. Test of simple main effects revealed no main effect of Distraction for the first- and last-5 words of each list (*F* < 1 in both cases), but a significant effect for the mid-list items, *F*(1,36) = 7.86, *p* = 0.008, partial η^2^ = 0.18, replicating the effect reported in Glenberg et al. (1998).

**FIGURE 3:**
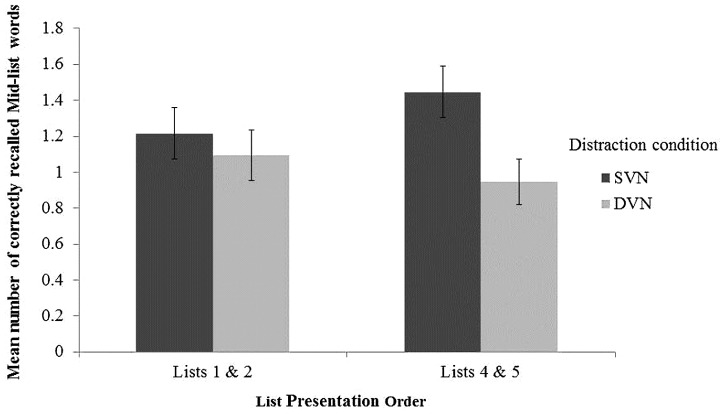
**The mean number of correctly recalled mid-list words from the first two- and last two- presented lists**. Error bars represent standard errors of the mean.

Having observed an effect of distraction for mid-list items, we conducted a follow up analysis exploring the effect of list-order on this effect. This looked at whether the effect of distraction increases across lists by comparing mid-list correct recall from the first-two and last-two lists presented under each distraction condition with a 2(Distraction: DVN vs. SVN) × 2(List Position: first two lists vs. last two lists) repeated measures ANOVA on mid-list recall. There was no main effect of list position, *F*(1,36) = 0.096, MSE = 0.634, *p* = 0.76, partial η^2^ = 0.003 but a main effect of distraction, *F*(1,36) = 6.11, MSE = 0.585, *p* = 0.018, partial η^2^ = 0.15 and a numerical trend towards an interaction of distraction with list position, *F*(1,36) = 3.46, MSE = 0.38, *p* = 0.071, partial η^2^ = 0.09, as illustrated in **Figure 3**. Simple main effects analysis revealed that the distraction effect was not significant for the first two lists (*F* < 1) but was significant for the last two lists, *F*(1,36) = 9.25, *p* = 0.004.

The final analysis looked at intrusion rates across lists. Because Glenberg et al. (1998) analysis was restricted to mid-list items, they did not look at intrusion rates because those could not be attributed to mid-list positions. Thus, their previously reported effect could have been due, in part, to distraction decreasing willingness to report (i.e., a criterion shift) rather than poorer memory. However, this did not appear to be the case here because there was a marginal increase in the rates of intrusions under distraction (DVN, *M* = 3.51, *SD* = 2.88; SVN, *M* = 2.70, *SD* = 2.16), *t*(36) = 1.96, *p* = 0.06, suggesting that distraction acts to reduce memory, rather than decreasing willingness to report.

Thus, we were successful in replicating the observed effect of distraction of mid-list items previously reported by Glenberg et al. (1998), using semantically neutral distraction. Additionally, distraction tended to increase error rates, in line with a memory deficit, rather than to reduce willingness to report. At first glance, these data appear to support the theoretical position advocated by Glenberg et al. (1998) and widely cited since, that visual distraction impairs moderately difficult recall. However, the other analyses challenge this theoretical position. First, overall recall for the full lists was not impaired by distraction: only memory for mid-list items. Therefore, the effects of distraction appear to be selective, rather than impairing memory generally. Second, the analyses of the different thirds of the list suggest that difficulty, as indexed by performance in the SVN condition, does not predict the likelihood of detecting a distraction effect. In particular, the final list items were as hard to recall as the mid-list items, consistent with our use of a post-list filler task to remove recency effects (Postman and Phillips, 1965) but showed no distraction effect. Finally, the analysis of order suggests that the overall effect for the mid-list items increases as interference increases across lists, with no distraction effect apparent for the earlier lists studied. This pattern is also inconsistent with the claim that distraction affects difficult recall, because greater distraction effects were found at the end of the lists, when mid-list recall was higher under SVN.

Whilst Experiment 1 was able to replicate the pattern reported by Glenberg et al. (1998), the overall pattern of findings is not consistent with the idea that visual distraction produces general memory impairment, or even an impairment that particularly affects difficult-to-recall items. There is a suggestion that the effect might be related to the build-up of interference over multiple lists, first because both the original demonstration, and our own, occurred in conditions in which multiple similar lists were studied, but also because the effect appeared to increase across lists. However, this is not compelling, because of the within-subject manipulation of distraction type, which meant that the first list of a particular condition was not necessarily the first list studied. For instance, a participant may have recalled the first list under EC instructions, the second under DVN, and the third under SVN. Each of these would be the first list in each condition, but the amount of interference would not be equal. Consequently we decided to run two further studies in which we explore two potential reasons why mid-list items might be susceptible to distraction in a multiple list paradigm.

## EXPERIMENT 2

Whilst the lack of a difference between the mid- and final-list items suggests that the difficulty of retrieval was not key to the distraction effect observed, this is not definitive because the argument rests upon a null effect. Consequently we decided to explore difficulty using a different manipulation. An alternate method for reducing the quality of memories to be retrieved is to impair their encoding. Consequently, in Experiment 2 we manipulated the presentation rate of the items. Participants either had 2 s per item (as in Experiment 1), or 0.5 s per item, with the clear expectation from the memory difficulty hypothesis that these items would be harder to recall, and so more susceptible to distraction.

The second potential explanation for the effects of distraction on mid-list items stems from the observation that the effect was stronger for later lists. The standard explanation for poorer recall with multiple lists is that there is a build-up of pro-active interference (Keppell and Underwood, 1962), such that the later lists become increasingly difficult to distinguish from previous lists. Thus, a possible modification of the vulnerable memory hypothesis is that distraction impairs the ability to distinguish between competing memories: thus, distraction does not impair recall when there is little competition, but it does so as the trials progress. In order to explore this idea we wanted to have greater control of the order of presentation of lists in each condition. Consequently we moved to a between-subjects manipulation of distraction, so that we could look at performance on the first list under each distraction condition, free from any potential interference from a previous list recalled under a different condition. We did not include the EC manipulation in this study.

A secondary prediction that derives from an account based upon interference is that the distraction effects across lists should be removed if the interference is reduced by a change of list structure. Consequently, Experiment 2 used the release from proactive-interference paradigm (Wickens et al., 1963; Loess, 1968), in which the first four successive lists all contained items from the same semantic categories, but the fifth list consisted of items from different categories. Thus, the interference account would predict increasing effects of distraction across the first four lists, but less distraction effect for the fifth list. Of course if list order *per se* (rather than interference) was key to the effect previously seen in Experiment 1, perhaps as a result of fatigue or loss of motivation as the study progressed, then the distraction effect would be expected to grow for list five, not reduce.

### METHOD

#### Participants

Sixty-four participants (38 females), average age 24.6 years (*SD* = 10.02) took part for course credit or as a paid volunteer.

#### Materials

In order to counterbalance the lists, we needed to move from 15- to 16-item word-lists. Ten 16-word high structured word-lists were created for this experiment from exemplars from 16 categories from Van Overschelde et al. (2004) semantic association norms. These were used to create two sets of five lists, both consisting of four interference lists (lists 1–4) and a release from interference list (list 5). Each interference list consisted of four exemplars from four different semantic categories (e.g., four professions, four fruits, four kinds of furniture, four animals). The fifth list consisted of four exemplars each from a different set of four categories. This process was repeated to create a second set of five lists, using different categories. For each participant, allocation of categories and items to list were randomly selected without replacement from the set of 16 categories. Mid-list items were defined as the middle six items, rather than five, with scores adjusted (by 5/6) when compared across list portions.

#### Procedure

The same basic procedure to Experiment 1 was followed, with participants studying and verbally recalling 10 successive lists, with the same filler task between study and test and participants unable to see the experimenter’s face throughout encoding and recall. Unlike Experiment 1, participants always received the same distraction condition during the retrieval period, either SVN or DVN. Additionally there was a manipulation of presentation rate. Participants studied five consecutive word-lists with words presented for 0.5 s each (fast presentation) and five word-lists with words presented for 2 s (slow presentation), counterbalanced for order across participants.

### RESULTS AND DISCUSSION

Experiment 2 was designed to explore two possible explanations for why DVN in Experiment 1 led to impaired mid-list recall of multiply presented lists: mid-list words are poorly encoded relative to the rest of the word-list; mid-list words are more susceptible to list inference than words in the rest of the list and either or both of these issues render mid-list recall vulnerable to distraction. In order to investigate these possibilities, we manipulated word presentation rate and list interference. We anticipated that presentation rates of 0.5 s vs. 2 s per word would lead to poorer encoding and therefore poorer recall and that repeatedly presenting same semantic category words across lists one to four (with a change in category for list five) would lead to a build-up of inter-list interference. In order to test the success of these manipulations, analysis first looked at the effect of presentation rate and list position (1–5) on overall correct recall.

#### Correct recall

The first analysis looked at correct recall, and the means are reported in **Table 1**. We ran a 2(Presentation rate: 0.5 s vs. 2 s) × 3(Word Position: first, mid, last items) × 5(List order: 1–5) × 2(Distraction: DVN vs. SVN) mixed ANOVA with repeated measures on all but the last factor. Overall, recall was better for slower presentation rates, *F*(1,62) = 194.2, MSE = 1.22, *p* < 0.001, partial η^2^ = 0.76, was poorer for mid-list items than at other list positions, *F*(2,124) = 8.41, MSE = 1.49, *p* < 0.001, partial η^2^ = 0.12, and showed a linear drop in correct recall across lists one to four, *F*(1,62) = 112.45, MSE = 3.1, *p* < 0.001, partial η^2^ = 0.65 coupled with a significant increase in recall from list four to five, *F*(1,62) = 27.64, MSE = 3.48, *p* < 0.001, partial η^2^ = 0.31.

**Table 1:** The mean number of correctly recalled words under SVN and DVN for lists 1–5, with fast and slow presentation rates.

		SVN						DVN					
		First	*SE*	Mid	*SE*	Last	*SE*	First	*SE*	Mid	*SE*	Last	*SE*
Fast presentation	List 1	1.31	*0.20*	1.17	*0.17*	1.47	*0.18*	2.00	*0.20*	1.30	*0.17*	1.69	*0.18*
	List 2	1.19	*0.20*	0.78	*0.17*	1.22	*0.17*	1.44	*0.20*	1.25	*0.17*	1.22	*0.17*
	List 3	1.19	*0.20*	1.07	*0.15*	0.81	*0.15*	1.22	*0.20*	0.78	*0.15*	1.06	*0.15*
	List 4	0.97	*0.18*	1.02	*0.17*	0.94	*0.18*	1.09	*0.18*	0.73	*0.17*	1.00	*0.18*
	List 5	1.16	*0.19*	0.83	*0.16*	1.28	*0.18*	1.44	*0.19*	1.17	*0.16*	1.34	*0.18*
														
Slow presentation	List 1	2.56	*0.24*	2.06	*0.17*	2.38	*0.23*	2.50	*0.24*	2.37	*0.17*	2.31	*0.23*
	List 2	1.97	*0.20*	1.72	*0.19*	1.66	*0.22*	2.25	*0.20*	1.80	*0.19*	1.88	*0.22*
	List 3	1.88	*0.23*	1.38	*0.18*	1.59	*0.22*	1.59	*0.23*	1.46	*0.18*	1.75	*0.22*
	List 4	1.72	*0.23*	1.25	*0.18*	1.25	*0.17*	1.50	*0.23*	1.51	*0.18*	1.69	*0.17*
	List 5	2.00	*0.25*	1.74	*0.20*	1.84	*0.23*	2.16	*0.25*	2.16	*0.20*	2.31	*0.23*

*Standard errors of the mean (*SE*) in italics.*

Given that our manipulations produced the expected effects on recall, the effect of Distraction was unexpected. Overall, more correct items were recalled under DVN than SVN, *F*(1,62) = 4.14, MSE = 11.15, *p* = 0.046, partial η^2^ = 0.063. Furthermore, Distraction did not reliably interact with any of the other factors in any combination (all *p*s > 0.16), and nor were there any other interactions (all *p*s > 0.093).

#### Incorrect recall

We ran a 2(Presentation rate: 0.5 s vs. 2 s) × 5(List order: 1–5) × 2(Distraction: DVN vs. SVN) mixed ANOVA on intrusion errors with repeated measure on all but the last factor, and the means are reported in **Table 2**. Overall the same number of incorrect words were given regardless of Presentation rate *F*(1,62) = 2.79, MSE = 0.41, *p* = 0.10, partial η^2^ = 0.043, but there was a List order effect *F*(2.81,174.53) = 12.036, *p* < 0.001, MSE = 0.675, partial η^2^ = 0.163 where repeated contrasts show that incorrect responses progressively increased from lists one to four but decreased for list five. More errors were produced under DVN than SVN, *F*(1,62) = 6.43, MSE = 0.27, *p* = 0.014, partial η^2^ = 0.094, but there was no interactions between Distraction and Presentation rate, or List order (*F* < 1 in all cases).

**Table 2:** The mean number of incorrectly recalled words under SVN and DVN for lists 1–5, with fast and slow presentation rates.

		SVN		DVN	
		Mean	*SE*	Mean	*SE*
Fast presentation	List 1	0.19	*0.09*	0.25	*0.09*
	List 2	0.47	*0.13*	0.59	*0.13*
	List 3	0.56	*0.17*	0.84	*0.17*
	List 4	0.47	*0.16*	0.91	*0.16*
	List 5	0.22	*0.09*	0.38	*0.09*
					
Slow presentation	List 1	0.13	*0.1*	0.31	*0.1*
	List 2	0.25	*0.13*	0.53	*0.13*
	List 3	0.5	*0.14*	0.69	*0.14*
	List 4	0.44	*0.19*	0.81	*0.19*
	List 5	0.06	*0.09*	0.31	*0.09*

*Standard errors of the mean (*SE*) in italics.*

#### First-list performance

Our original intention was to examine the nature of any overall distraction effect specifically for the first list. In line with the overall analyses, there were no effects of Distraction, nor any interactions involving Distraction, on correct recall or intrusions. To save space we do not report them here but full details are available from the first author on request.

Thus, in summary, although the manipulations of presentation rate and list interference manipulations were successful in moderating recall performance, they did not interact with the effects of distraction. Moreover, the main effects of distraction did not replicate that found in Experiment 1. Whilst distraction once again increased errors, it also increased correct recall. In fact, it appeared that the magnitude of the effects on correct and incorrect recall was approximately the same, with an increase of Cohen’s *d* = 0.54 in correct recall, and Cohen’s *d* = 0.63 for errors. Thus, despite the increase in errors, there is little evidence to support the idea that DVN causes impairment of memory, but rather that it shifts willingness to report an answer that comes to mind. These patterns were not moderated by position of the words in the list. Thus these data do not appear to be consistent with inter-list interference and poor encoding as explanations for the distraction effect seen for mid-list items in Experiment 1 and seen in Glenberg et al. (1998) study.

One difference between the studies that showed an impairment of recall from distraction, and Experiment 2 is that the previous studies used entirely unstructured lists containing unrelated items both within- and across-lists. In contrast, Experiment 2 used list structure as a means of manipulating interference, and consequently used a restricted set of items. One possibility is that participants utilized this structure in their retrieval strategies and were able to overcome any environmental distraction. Consequently we looked at the role of list structure in Experiment 3, whilst controlling for item effects.

## EXPERIMENT 3

In Experiment 2, interference came from both inter-list repeated categories and intra-list same-category words. That is, for a particular participant, each of the first 4 lists contained multiple exemplars from the same categories. So, although participants were clearly affected by the build-up of list interference (correct recall decreased across each set of lists 1–4 and incorrect recall increased), they may have adopted a recall strategy that used their knowledge of the list structure (i.e., the semantic categories contained in each list) which made them less susceptible to the negative effects of distraction. This experiment manipulated the degree of list structure (and cross-list similarity) whilst controlling for item effects by repeatedly sampling the same pool of 16 items from 16 categories. In the high structure condition, participants saw four exemplars from four categories successively for four lists, repeating this (with different categories) four times overall. In contrast, the low structure condition saw one exemplar from each of the 16 categories for 16 trials. Thus, across all lists, both conditions were matched for the items studied. However, the high structure condition resembled the structure used in Experiment 2, with the expectation that we would observe build-up of proactive-interference across the sets of four lists (with release from interference between sets). In contrast, the low structure condition resembled Experiment 1, in that the lists were as unstructured as they could be, given the constraint that the same set of items was used. If structure is the key difference between the first two studies, we expected to see a greater distraction effect for the unstructured condition than for the structured condition.

### METHOD

#### Participants

Thirty-six participants (23 females), average age 22.6 years (*SD* = 8.86) took part for course credit or as a paid volunteer.

#### Materials

The same 16 category word-lists used in Experiment 2 were used to create a set of 16 high and 16 low structured word-lists, each consisting of 16-words. High structured lists were created in the same way as experimental lists one to four in Experiment 2, and thus constituted lists for which interference was expected to build-up over the four lists. Low structured lists were created by randomly selecting, without replacement, one word from each of the 16 category word-lists.

#### Procedure

Participants studied and then recalled either 16 high or 16 low structured-lists, under the same conditions as Experiment 1. The nature of the distraction was held constant for blocks of four lists, and then switched, with this repeated until all 16 lists had been tested, with participants recalling eight lists under DVN and eight under SVN, with order counterbalanced across participants. Otherwise, the experimental conditions replicated Experiment 2.

### RESULTS AND DISCUSSION

Experiment 3 manipulated inter- and intra- list structure: we anticipated that high structured lists would build-up inter-and intra-list interference and impair recall (as was found in Experiment 2) to a progressively greater degree across lists one to four than low-structured lists. Therefore the analyses presented below for both full-list and mid-list correct and incorrect recall begins by seeking to confirm the success of this manipulation before looking at any effect of distraction on recall.

#### Correct recall

The first analysis looked at correct recall, and the means are reported in **Table 3**. We ran a 2(List structure: low vs. high) × 3(Word Position: first, mid, last items) × 5(List order 1–4) × 2(Distraction: DVN vs. SVN) ANOVA with repeated measure on all but the first factor. Overall, low-structured lists were recalled as well as high structured lists, *F*(1,34) = 1.64, MSE = 8.39, *p* = 0.21, partial η^2^ = 0.046, but recall was poorer for mid-list items than for other list-position items, *F*(2,57.16) = 10.55, MSE = 1.11, *p* < 0.001, partial η^2^ = 0.23. There was a linear drop in correct recall across lists one to four, *F*(3,93.12) = 10.02, MSE = 0.53, *p* < 0.001, partial η^2^ = 0.23 with a one-way ANOVA on List order showing a linear decline for High structured lists, *F*(1,68) = 15.58, *p* < 0.001, η^2^ = 0.21, but no such effect for Low structured lists, *F*(1,68) = 0.51, *p* = 0.48, η^2^ = 0.013.

**Table 3:** The mean number of correctly recalled First-, Mid-, and Last-list words under SVN and DVN for high- and low-structured lists 1–4.

		SVN						DVN					
		First	*SE*	Mid	*SE*	Last	*SE*	First	*SE*	Mid	*SE*	Last	*SE*
High structured	List 1	2.75	*0.27*	1.83	*0.21*	2.08	*0.22*	2.08	*0.30*	2.01	*0.21*	2.28	*0.23*
	List 2	2.11	0.27	2.00	0.22	1.75	0.24	2.39	0.27	2.09	0.23	1.89	0.21
	List 3	1.72	*0.21*	1.44	*0.19*	1.81	*0.23*	2.14	*0.32*	1.53	*0.22*	1.75	*0.26*
	List 4	1.75	*0.27*	1.67	*0.25*	1.81	*0.25*	1.56	*0.27*	1.30	*0.19*	1.83	*0.24*
													
Low structured	List 1	2.03	*0.27*	1.46	*0.21*	1.94	*0.22*	1.78	*0.30*	1.60	*0.21*	1.86	*0.23*
	List 2	1.92	*0.27*	1.30	*0.22*	1.61	*0.24*	1.78	*0.27*	1.48	*0.23*	1.56	*0.21*
	List 3	1.94	*0.21*	1.48	*0.19*	1.50	*0.23*	1.81	*0.32*	1.27	*0.22*	1.69	*0.26*
	List 4	1.44	*0.27*	1.20	*0.25*	1.75	*0.25*	2.00	*0.27*	1.20	*0.19*	1.89	*0.24*

*Standard errors of the mean (*SE*) in italics.*

Although our manipulations produced the expected effects on recall, there was no overall main effect of Distraction, *F* < 1and no interactions involving Distraction at all (all *p*s > 0.10).

#### Incorrect recall

We ran a 2(List structure: low vs. high) × 4(List order 1–4) × 2(Distraction: DVN vs. SVN) mixed ANOVA on intrusion errors with repeated measure on all but the first factor, and the means are reported in **Table 4**. Overall, progressively more incorrect words were recalled across lists one to four, *F*(2.3,78) = 5.22, MSE = 0.20, *p* = 0.005, partial η^2^ = 0.13, however, low- and high- structured lists did not differentially affect incorrect recall, *F* < 1. There was no main effect of Distraction, *F*(1,34) = 1.61, MSE = 0.24, *p* = 0.21, partial η^2^ = 0.05 and there were no interactions involving Distraction (all *p*s > 0.28).

**Table 4:** The mean number of correctly recalled words under SVN and DVN for high- and low-structured lists 1–4.

		SVN		DVN	
		Mean	*SE*	Mean	*SE*
High structured	List 1	0.25	*0.14*	0.25	*0.12*
	List 2	0.47	*0.12*	0.61	*0.11*
	List 3	0.56	*0.13*	0.58	*0.12*
	List 4	0.86	*0.16*	0.56	*0.14*					
Low structured	List 1	0.69	*0.14*	0.36	*0.12*
	List 2	0.42	*0.12*	0.33	*0.11*
	List 3	0.39	*0.13*	0.44	*0.12*
	List 4	0.61	*0.16*	0.53	*0.14*

*Standard errors of the mean (SE) in italics.*

In short, this study found no reliable effects of distraction at all, despite once again demonstrating list position effects, and interference effects. Therefore the absence of a distraction effect in Experiment 2 does not appear to be a result of the high level of structure used in that Experiment. This does not rule out the possibility that the absence of evidence of an effect (and the presence of the effect in previous studies) reflects some unknown attributes of the items, because Experiment 3 used the same pool of items as Experiment 2, which was different from the set used for Experiment 1. However, whilst we cannot rule out this possibility, it does leave the theoretical explanation of the effect with little explanatory power, because any account would require that the negative effects of environmental distraction appears to occur only for particular items, studied as mid-list items of multiple lists.

## GENERAL DISCUSSION

The main purpose of the studies was to investigate whether Glenberg et al.’s (1998) findings (Experiment 5) could be replicated, that is, whether visual distraction impairs verbal recall. Experiment 1 did find a moderately sized distraction effect for recall of the mid-list items, but this pattern was not replicated in either Experiment 2 or Experiment 3. Moreover, looking at data from the full word-lists presents a consistent negative picture. When analyzing memory for all the items in the list, there was no evidence of distraction impairing correct recall, whilst Experiment 2 showed that DVN *increased* full-list correct recall, albeit with a concomitant increase in errors. Results for incorrect recall were less consistent. Distraction had no effect on incorrect recall in Experiment 1 or Experiment 3 but, increased errors for multiple lists in Experiment 2.

The results of Experiments 2 and 3 clearly show effects of word presentation rate, interference and word position on recall; as the task became more demanding participants recalled fewer correct words and made more errors. Approximately one third of words were recalled from each word-list with no obvious floor or ceiling effects restricting our ability to detect an effect of distraction. Therefore, if visual noise competes with demanding retrieval processes for finite resources we would expect to have seen an effect on one of the tasks presented but we did not.

**FIGURE 4:**
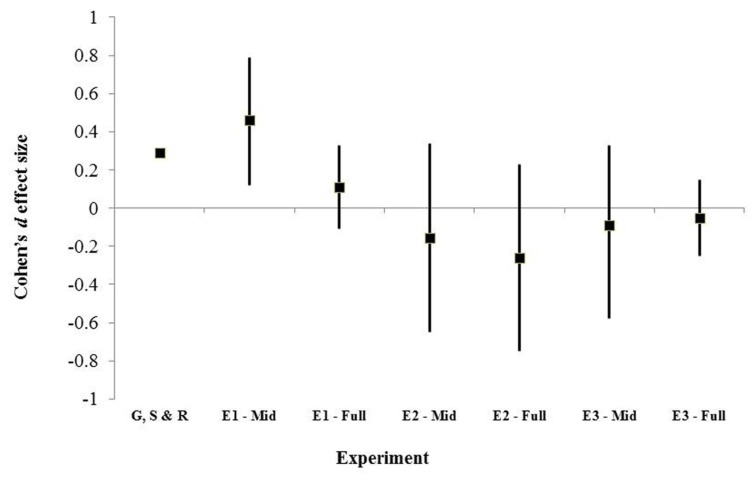
**The mean effect sizes (Cohen’s d) and 95% confidence intervals for Mid-list and Full-list correct recall under DVN for Experiments 1 to 3 (E1–E3).** Glenberg et al.’s (1998; G,S and R) mean effect size is included for comparison, but no confidence intervals are available.

**Figure 4** illustrates the overall pattern for the studies reported here, both for recall of mid-list items, and for recall of all items. This plots mean effect size and 95% confidence intervals around those effect sizes for each study. Glenberg et al.’s (1998) mean effect size is included for comparison, but no confidence intervals are available. This illustrates that five out of six potential effect sizes are compatible with their being no effect. The more optimistic reading of these data is that all studies are compatible with a very small effect: the confidence intervals calculated for each study all include the range *d* = 0.12 to *d* = 0.15. Thus, the appropriate conclusion to be drawn from the current series of studies is that there is either no impact of distraction upon recall from word-lists, or very little effect, irrespective of the difficulty of the memory materials.

This forces us to reconsider the central claim upon which the memory distraction effect was predicted: that the environment competes for cognitive resources with internal processing to the detriment of recall. In our studies, participants engaged in extensive and difficult memory retrieval tasks, for multiple lists of similar words presented at a fast rate. Performance was well below ceiling and so could be regarded as moderately demanding memory tests. At the same time our visual distraction condition required participants to look directly at a screen containing flickering images, modeled on previous studies that have demonstrated that such images are distracting to cognitive performance (McConnell and Quinn, 2000; Andrade et al., 2002; Quinn and McConnell, 2006; Dean et al., 2008; Parker and Dagnall, 2009). And yet we observed little, if any effect on recall.

We cannot rule out the possibility that other forms of distraction or other forms of memory test might have produced a distraction effect. For example, it is feasible that word-list recall involves a comparatively large semantic component and a relatively small visual component. Thus a visual distractor which engages the semantic system (such as a film-clip) may affect recall more than a visual distractor that does not engage the semantic system (such as black and white squares). However, this explanation is not supported by Perfect et al. (2012) who found a strong effect of a semantically neutral visual distractor (colored boxes) on recall of auditory as well as visual details. Anecdotally we can report that we have run many attempts in our laboratory to find evidence for distraction effects on memory for word-lists, but without success. But the fact that a distraction effect is *possible* misses the point that the effect is not *inevitable*, and thus challenges the central tenet of the theoretical claim that environmental distraction competes for resources with internal processing resources during recall. The question is not whether environmental distraction does or does not produce an impairment of recall – because both have been shown – but under what conditions environmental distraction impairs recall. What needs explanation is why studies of event memory report moderate to large effect sizes for the negative effects of distraction and the positive effects of EC to reduce distraction, but the studies using memory for lists appear to show little, if any effect. Currently, we cannot offer a definitive reason for this distinction, but in the final section we offer a speculative account, based on an interesting study from the eyewitness field.

One possible explanation for the differential effect of distraction on event memory and memory for word-lists could be the role of contextual reinstatement (for a review see Smith, 2013). If mental reinstatement is used as a search strategy to retrieve details of episodic events, the richness of context information available for word-lists may be far diminished compared to that for events. Mentally reinstating a word-list, such as the ones presented in the experiments here, involves reinstating a white computer screen with black print at its center; there is very little context here to associate the word to, each word is presented on the same white screen so there are scarcely any other central contextual cues with which to discriminate each word from another. In this case, mental reinstatement will provide little benefit and semantic associations made at encoding may overshadow encoding of the impoverished central contextual environment. Likewise, the focus on semantic associations at retrieval may outshine or overpower the impoverished contextual cues. The result is that the physical central context may play a relatively small role in encoding and retrieving word-list items. On the other hand, mentally reinstating an event is rich with contextual cues within the source memory itself and as a result, the contextual cues from the event itself may be crucial in the recall of details from the event. Thus, an intriguing possibility is that distraction interferes with mental context reinstatement specifically. That is, the current environment can interfere with the ability to reconstruct a past context, rather than the ability to directly access memories. Thus, memories that benefit from the recreation of a past context (i.e., complex event details) are hindered by distraction whilst memories that can be accessed without context cues (i.e., semantic tokens presented in a sparse context) are not.

Consistent with this view, Vredeveldt and Penrod (2013) recently looked at the interaction between distraction reduction through EC, and context reinstatement. All participants witnessed an event in a busy street. Half were then interviewed in that street with lots of on-going distraction, and half in a quiet office, with little distraction. In each case, half had their eyes-closed. The hypothesis that environmental distraction competes with recall would predict that EC would be most beneficial in the busy street, but it was not. It was of most use in the quiet office, when witnesses had changed their retrieval context. Thus, even a quiet environment can be distracting if it conflicts with the ability to reconstruct the appropriate retrieval context, and a distracting New York city street can be non-distracting if it supports memory by providing useful cues.

## Conflict of Interest Statement

The authors declare that the research was conducted in the absence of any commercial or financial relationships that could be construed as a potential conflict of interest.
